# Determining the role of sarcomeric proteins in facioscapulohumeral muscular dystrophy: a study protocol

**DOI:** 10.1186/1471-2377-13-144

**Published:** 2013-10-11

**Authors:** Saskia Lassche, Coen AC Ottenheijm, Nicol C Voermans, Henk-Jan Westeneng, Barbara H Janssen, Silvère M van der Maarel, Maria T Hopman, George W Padberg, Ger JM Stienen, Baziel GM van Engelen

**Affiliations:** 1 Department of Neurology, Radboud university medical center, Nijmegen, The Netherlands; 2 Department of Physiology, Institute for Cardiovascular Research, VU University Medical Center, Amsterdam, The Netherlands; 3 Department of Neurology, University Medical Center Utrecht, Utrecht, the Netherlands; 4 Department of Radiology, Radboud university medical center, Nijmegen, the Netherlands; 5 Department of Human Genetics, Leiden University Medical Center, Leiden, the Netherlands; 6 Department of Physiology, Radboud university medical center, Nijmegen, the Netherlands

**Keywords:** Facioscapulohumeral muscular dystrophy, Oculopharyngeal muscular dystrophy, Sporadic inclusion body myositis, Skeletal muscle, Sarcomere, Myofilament

## Abstract

**Background:**

Although muscle weakness is a hallmark of facioscapulohumeral muscular dystrophy (FSHD), the molecular mechanisms that lead to weakness in FSHD remain largely unknown. Recent studies suggest aberrant expression of genes involved in skeletal muscle development and sarcomere contractility, and activation of pathways involved in sarcomeric protein degradation. This study will investigate the contribution of sarcomeric protein dysfunction to the pathogenesis of muscle weakness in FSHD.

**Methods/Design:**

Evaluation of sarcomeric function using skinned single muscle fiber contractile studies and protein analysis in muscle biopsies (quadriceps femoris and tibialis anterior) from patients with FSHD and age- and gender-matched healthy controls. Patients with other forms of muscular dystrophy and inflammatory myopathy will be included as disease controls to assess whether results are due to changes specific for FSHD, or a consequence of muscle disease in general. A total of 56 participants will be included. Extensive clinical parameters will be measured using MRI, quantitative muscle studies and physical activity assessments.

**Discussion:**

This study is the first to extensively investigate muscle fiber physiology in FSHD following an earlier pilot study suggesting sarcomeric dysfunction in FSHD. The results obtained in this study will increase the understanding of the pathophysiology of muscle weakness in FSHD, and possibly identify novel targets for therapeutic intervention.

## Background

Facioscapulohumeral muscular dystrophy (FSHD) is the third most common hereditary myopathy, affecting 1:15,000-20,000 people [1]. Age of onset and disease severity is variable, although in general presentation starts in the second decade of life [2]. FSHD derives its name from the characteristic early involvement of the muscles of the face, shoulder girdle and upper arm, which, in contrast to other muscular dystrophies, is often asymmetrical. Further in the disease course weakness progresses to the foot dorsiflexors, abdominal and proximal leg muscles [3]. About 20% of patients become wheelchair dependent [4].

FSHD1 (OMIM #158900), the most common type of FSHD, is an autosomal dominant disorder caused by a contraction of the D4Z4 repeat array, a macrosatellite repeat array consisting of 3.3 kb large D4Z4 units located on chromosome 4q. FSHD patients have 1 – 10 repeat units, in contrast to unaffected individuals who have 11 – 100 units [5,6]. Residual repeat length is roughly and inversely correlated to disease severity and onset. Patients having 1 – 3 repeats usually show infantile onset and rapid disease progression [7]. The repeat array contraction leads to a more open D4Z4 chromatin configuration, which is hypothesized to permit transcription of otherwise epigenetically silenced genes located on chromosome 4 [5,6]. For FSHD to develop, there also needs to be a permissive genetic background of chromosome 4, as described below. In a minority of patients with a FSHD phenotype there is no D4Z4 contraction. These patients are categorized as FSHD2 and the majority of them have a mutation in the chromatin modifier SMCHD1 [8,9]. Both FSHD1 and FSHD2 share the above-mentioned changes in D4Z4 chromatin configuration in somatic tissue on a FSHD-permissive chromosome 4 background.

FSHD candidate genes include Double Homeobox Protein 4 (*DUX4*) and FSHD Region Gene 1 (*FRG1*). Permissive chromosome 4 backgrounds contain a polymorphic *DUX4* polyadenylation signal, which facilitates ectopic expression of DUX4 in skeletal muscle upon D4Z4 chromatin opening. DUX4 is currently considered the primary mediator of FSHD pathology, and activates the p53 and caspase-3 pathways when overexpressed in mice. Both of these pathways are involved in skeletal muscle differentiation, sarcomeric protein degradation and apoptosis [10,11]. DUX4 may also activate ubiquitin-mediated protein degradation pathways including E3 ubiquitin ligases such as Atrogin-1 and MuRF1 [12,13]. These muscle specific ligase enzymes specifically target sarcomeric proteins and have been implicated in the atrophic phenotype in FSHD myotubes [13,14]. FRG1 is localized to the Z-disc of the sarcomere and functions as an actin-binding and bundling protein [15,16]. Overexpression of FRG1 in mice causes muscular dystrophy and vascular abnormalities, both features of FSHD [17,18]. Taken together, these findings suggest that expression of FSHD candidate genes such as *DUX4* or *FRG1* have downstream effects on sarcomere development and sarcomere turnover.

The sarcomere – the smallest contractile unit in muscle – is composed of several proteins that work together to enable muscle contraction. Upon activation with calcium, cross-bridges are formed between actin and myosin filaments. This induces filament sliding and enables muscle contraction. Other important constituents of the sarcomere include titin, which stabilizes the sarcomere and contributes to passive force, and nebulin, which plays an important role in the regulation of actin filament length.

We hypothesize that dysfunction of sarcomeric proteins contributes to the pathogenesis of muscle weakness in FSHD. A pilot study in 4 FSHD muscle biopsies performed in our laboratory supports this hypothesis [19]. The present study aims to confirm these data in a larger cohort and elucidate the causes of sarcomeric dysfunction in FSHD. Secondary objectives are:

– To determine whether sarcomeric dysfunction is specific for FSHD, or part of a generalized pathology common to muscular dystrophy and/or inflammatory myopathy. To investigate this, patients with oculopharyngeal muscular dystrophy (OPMD) and sporadic inclusion body myositis (sIBM) will be included in the study as disease controls.

– To explore other contractile properties of FSHD muscle fibers such as cross-bridge cycling kinetics, calcium sensitivity and passive force generation. With this, we aim to clarify the cause of sarcomeric dysfunction in FSHD.

– Compare sarcomeric function in muscles affected early (tibialis anterior) and late (quadriceps femoris) in the disease course of FSHD to shed light on the remarkable distribution of weakness in FSHD. Also, by assessing these muscles within the same patient we will be able to determine when sarcomeric dysfunction occurs in the pathological process.

– Correlate *in vitro* sarcomeric function to *in vivo* clinical parameters such as physical activity, muscle strength, disease severity, residual D4Z4 fragment length, muscle/fat fraction and quantitative muscle studies.

– This study will also provide extensive new data on sarcomeric function in OPMD and sIBM. This will result in a better understanding of these disorders and generate new hypotheses for research and treatment.

## Methods/Design

### Study population

Genetically confirmed patients with FSHD1 will be recruited from the Radboud university medical center. Healthy individuals without a history of neuromuscular disease will be included as healthy controls. Two disease control groups (one with muscular dystrophy and one with inflammatory myopathy) will be included to determine whether the results obtained in this study are specific for FSHD, or a general feature of muscular dystrophy or inflammation – both histological features of FSHD. For the muscular dystrophy disease control group, patients with genetically confirmed OPMD (12–17 trinucleotide repeats in *PABPN1*) will be included. OPMD was chosen because it is a relatively common muscular dystrophy, has an adult age of onset and has no associated systemic features which might influence muscle fiber function. Also, patients are not likely to be wheelchair bound, which is an exclusion criterion for this study. For the inflammatory myopathy control group, patient with sIBM fulfilling the modified 2010 Griggs criteria will be included [20]. We have chosen sIBM because it is relatively common, has clearly delineated histological features and diagnostic criteria, and patients are not treated with corticosteroids, which is an exclusion criterion for this study. The full list of exclusion criteria is as follows:

– Age <18 or >65 years

– Diabetes mellitus

– Chronic obstructive pulmonary disease

– Chronic heart failure

– Current malignancy

– Previous treatment with chemotherapy and/or radiation therapy

– Use of corticosteroids during more than two weeks in the past 5 years

Use of statins in the past year

– Wheelchair bound

Contra-indications for MRI or muscle biopsy

– Pregnancy

In each group, 14 participants will be included for a total of 56 participants. Age and gender matching will be applied on the group level. The Medical Ethics Review Committee region Arnhem-Nijmegen approved the study protocol.

### In vivo study procedures

#### General

Information on medical history and current medication use will be collected. Body weight and length will be measured. Parameters of muscle injury will be assessed in serum (creatine kinase (CK), lactic dehydrogenase (LDH), aspartate transaminase (AST) and alanine transaminase (ALT)). In FSHD patients, disease severity will be assessed using the Ricci Clinical Severity Scale (CCS) and the CSS corrected for age: age-corrected CSS (ACCSS) [21-23].

#### Physical activity and muscle strength

Physical activity will be assessed in several ways. Participants will be equipped with an actometer, a device that is attached to the ankle to constantly measure physical activity during 12 days [24,25]. Validated questionnaires will be administered to assess participants' daily living activities, disease impact and possible confounding factors such as pain: Health Assessment Questionnaire (HAQ) [26], International Physical Activity Questionnaire (IPAQ) [27], Sickness Impact Profile (SIP 68) [28], McGill’s Pain Questionnaire (MPQ) [29]. Muscle strength will be graded with the Medical Research Council (MRC) scale [30]. A trained examiner will obtain a Motor Function Measure [31]. In addition, a six-minute walk test and timed up and go test will be performed [32,33].

#### Quantitative muscle studies

Isometric force recordings of maximal voluntary and electrical evoked contractions of the quadriceps femoris will be performed as described previously [34]. Participants will be seated in a custom-build chair with the knee angle set at 120°. To prevent compensatory movements, participants are strapped at the hips and upper body to maintain their position. Electrical pulses will be delivered through two self-adhesive surface electrodes placed proximally over the rectus femoris and distally over the vastus medialis portion of the quadriceps muscle. Two gel electrodes will be used to make a surface electromyogram (sEMG).

##### Maximum voluntary contraction

All measurements will be performed in the leg from which the muscle biopsy will be taken (i.e., the right leg, unless in the presence of asymmetrical weakness in which case the weakest leg will be biopsied). Participants will be asked to perform a Maximum Voluntary Contraction (MVC) during 3 seconds. The highest force from three contractions is used to represent the MVC. Next, the 100 Hz current that evokes 30% of MVC will be determined and used for all subsequent measurements.

##### Contractile properties

After a 5-minute rest period, the force–frequency relation will be determined with 1 Hz, 100 Hz, 1 Hz, 10 Hz, 20 Hz, 30 Hz, and 50 Hz pulse trains of 1 s duration. There will be a 1-minute rest period after each contraction. The 1 Hz stimulation will be performed multiple times to verify that the 100 Hz pulse train causes no fatigue or potentiation. The contraction time, rate of force rise, and early and late relaxation times will be derived from the 100 Hz contraction (positively filtered with a 30 Hz filter frequency). These parameters provide a measure for the rate of cross-bridge cycling and the rates of Ca^2^ reuptake by the sarcoplasmic reticulum.

##### Fatigue

After another 5-minute rest period, a series of 30 Hz 1 s on 1 s off pulses will be applied for a period of 2 minutes to determine muscle fatigability. Fatigue is determined as the relative decline of force between the first three contractions compared with that of the last three contractions.

### MRI

A MR exam of the upper and lower leg will be performed in all participants using a 3 Tesla MR system (Tim TRIO, Siemens, Erlangen, Germany) with the spine array coil and two phased-array coils placed around the legs. A fish-oil marker will be placed on the site where the muscle biopsy will be taken from, to ensure that MRI findings correspond to the approximate site of muscle biopsy. Patients will be placed in the scanner feet first supine. The table will be positioned to have first the upper and subsequently the lower leg in the isocenter of the magnetic field for imaging of the thigh and calf muscles respectively.

Scout images will be acquired in three orthogonal directions for accurate positioning of the MRI slices. Thereafter 8 transversal slices (FOV 175×175 mm^2^, thickness 4 mm, gap 6 mm, baseresolution 256) will be acquired with a T2 multi spin echo sequence (TR: 3000 ms, 16 equally spaced echo times 7.7 – 123.2 ms). Subsequently followed by 23 transversal slices (thickness 4 mm, gap 0.4 mm) that will be obtained with a T1 turbo spin echo sequence (FOV 250×244.5 mm^2^, TR/TE 600 ms/13 ms, baseresolution 448), and with a Turbo Inversion Recovery sequence (TIRM) (FOV 175x175 mm^2^, TR/TE/TI 4100 ms/41 ms/220 ms, baseresolution 256). The same imaging protocol will be used for both the upper and lower leg.

Hyperintensities on the TIRM images will be detected visually as an indicator for edema and/or inflammation [35]. Muscle fraction at the slice corresponding to the approximate site of muscle biopsy will be quantified using the method described by Kan [36].

### In vitro study procedures

#### Muscle biopsies

One needle muscle biopsy of the quadriceps femoris (vastus lateralis), and one of the tibialis anterior will be obtained from each participant. The tibialis anterior is frequently affected in FSHD, but early involvement of the quadriceps femoris is uncommon [3,37]. In the presence of symmetrical muscle strength, the right side will be biopsied. In case of asymmetrical weakness based on neurological examination, the weakest muscle will be biopsied.

An experienced neurologist or neurology resident, taking routine antiseptic precautions, will perform the muscle biopsies. Biopsy material intended for histology will be frozen in isopentane and stored at −80°C. Material for protein analysis will be immediately frozen in liquid nitrogen and stored at −80°C. Specimens intended for contractile studies are stored at −20°C in a glycerinating solution containing half glycerol and half relaxing solution. For composition of this solution, see below.

#### Muscle fiber preparation

For contractile studies, biopsy material will be placed in a relaxing solution containing 1% (v/v) Triton X-100 at 4°C [38]. Triton is used to permeabilize the plasma membranes, resulting in “skinned” muscle fibers. The skinning process enables studies of sarcomeric function in isolation by eliminating the confounding effects of upstream processes of excitation-contraction coupling, such as sarcoplasmatic reticulum Ca^2+^ handling. Protease inhibitors will be added to the solution to prevent protein degradation. Skinned single muscle fibers will be isolated and fiber ends will be attached to aluminum t-clips. The clips are then mounted to a length motor on one end, and a force transducer on the other.

#### Muscle fiber mechanics

##### Maximum force generation and cross-bridge cycling kinetics

Skinned muscle fibers will be activated by sequentially bathing them in a relaxing solution, pre-activation solution and activation solution. The composition of these solutions has been described previously [39]. Force generating capacity will be measured at a sarcomere length of 2.5 μm. Maximal force generating capacity will be measured at pCa 4.5 and corrected for cross-sectional area. To determine whether changes in maximal force are due to changes in the kinetics of cross-bridge attachment and detachment, a rapid stretch-release protocol will be imposed on an activated fiber. The rate constant of tension redevelopment (K_tr_) will be determined by fitting the rise of tension to the following equation: F = F_ss_(1 – e^-Ktr·t^) [40]. After force redevelopment reaches the steady-state maximal force again, active stiffness will be determined. Length changes of sequentially 0.3, 0.6, 0.9, -0.3, -0.6 and −0.9% will be applied, during which peak force is measured. Corrected peak force will be linearly plotted against the imposed length changes, with the slope representing the fraction of strongly attached cross bridges [41].

##### Calcium sensitivity of force generation

During daily-life muscle activation, individual fibers typically function at a submaximal level. Therefore, the calcium sensitivity of force generation provides additional information on contractile properties. The calcium sensitivity of force generation will be measured by bathing skinned muscle fibers in activation solutions with different pCas ranging from 4.5-7.0 and measuring steady-state force. The obtained force-pCa data will be fitted to the Hill equation, providing the pCa_50_ (the pCa giving 50% of maximal active tension) and the Hill coefficient, n_H_ (an index of myofilament cooperativity) [42].

##### Passive force generation

Passive force generation will be measured by placing skinned muscle fibers in a chamber filled with relaxing solution and imposing length changes as previously described [43]. The fibers will be set to their slack length (i.e. the length at which passive force is zero) and from there they will be stretched with a constant velocity to a sarcomere length of 3.2 μm, held for 90 s and then released back to slack length. Tension development during stretch will be determined.

#### Muscle protein analysis

To study whether changes in sarcomere contractile function are a consequence of proteomic alterations, extensive protein analysis using SDS-PAGE and Western blotting will be performed as described previously [43,44]. We will pay special attention to myosin heavy chains, actin, titin, nebulin and regulatory proteins such as troponin and tropomyosin. In addition we will determine oxidation/nitrosylation (i.e. posttranslational modifications in response to oxidative stress) of sarcomeric proteins by Oxyblot and Western blotting, as described previously [45].

To study whether decreased maximal force generation is a result of decreased contractile protein content, we will determine myosin content per half sarcomere and will relate the results to the force generated by those fibers as described previously [46]. In brief, myosin heavy chain (MHC) content and MHC isoform composition will be determined using SDS-PAGE. We will calculate fiber myosin concentration by dividing total MHC content by fiber volume (at a sarcomere length of 2.5 μm). This fiber myosin concentration will be multiplied by half-sarcomere volume to calculate MHC content per half sarcomere. Finally, MHC content per half sarcomere will be related to muscle fiber mechanics.

For analysis of titin and nebulin exon composition, we will use a titin/nebulin exon microarray that consists of 50-mer oligonucleotides representing all exons in the titin gene, spotted in triplicate on the array. The array contains human titin and nebulin genes as well as all known titin-binding proteins [47].

#### Histology

Histology will be used for light-microscopic evaluation of frozen sections. We will study size, shape, type and cytoarchitecture of muscle fibers, presence of internalized nuclei, destruction of muscle fibers and regeneration, as well as supporting connective tissue.

### Outcome assessment

The primary outcome measurement of this study is the maximum force generating capacity (mN/mm^2^) of permeabilized single muscle fibers of FSHD patients and healthy control subjects. Secondary outcome measurements are:

– The maximum force generating capacity of OPMD and sIBM fibers in relation to FSHD fibers.

– Contractile properties of FSHD fibers such as cross-bridge cycling kinetics, calcium sensitivity and passive force generation in relation to fibers of healthy controls.

– Maximum force generating capacity and contractile properties of muscle affected early (tibialis anterior) compared to muscle affected late (quadriceps femoris) in the disease course.

– Analysis of sarcomeric proteins and titin and nebulin exon composition of FSHD muscle.

– *In vivo* clinical parameters such as physical activity, muscle strength, disease severity, residual D4Z4 fragment length, muscle/fat fraction and quantitative muscle studies.

### Statistical analysis and power calculation

#### Statistical analysis

Differences between the above-mentioned outcome measurements will be analyzed using analysis of covariance (ANCOVA). A p value <0.05 will be considered significant.

### Power

Due to the exploratory nature of this study, there are virtually no previous studies to refer to for power calculations. Based on our pilot study, we aim to test the hypothesis that there is a difference in maximum force generating capacity of 7 mN/mm^2^ (~10% of FSHD maximum force generation), with standard deviations of 6 mN/mm^2^, α = 0.05 and β = 0.20. This hypothesis requires a sample size of 13 subjects per group. Because the muscle tissue can be damaged during the biopsy procedure, we anticipate that ~10% of the collected biopsies will not be useful for experiments. This brings the total sample size to 14 subjects per group.

## Discussion

Over the past decades, the identification and understanding of the complex genetic and epigenetic mechanism underlying FSHD has been the primary focus of the scientific community. Recently, this effort resulted in a unifying genetic model for FSHD [5]. However, many questions concerning the molecular and physiological mechanisms underlying muscle weakness in FSHD remain unanswered. This study aims to look at the pathophysiology of FSHD from a different perspective, and is the first to extensively investigate the pathophysiology of muscle weakness in FSHD.

A major strength of this study is the combined analysis of extensive *in vitro* and *in vivo* data in the same cohort of patients and control subjects. This will provide the unique opportunity to increase our understanding of the relationship between pathophysiological mechanisms and the clinical condition of the patient. Biopsies of two muscles with either early or late involvement (tibialis anterior and quadriceps femoris, respectively) will be collected specifically for this study to provide insight into the consequences of disease progression and the distribution of weakness in FSHD. Studies of patients with other forms of muscular dystrophy and inflammatory myopathy will not only indicate whether the observed changes found are specific to FSHD or a consequence of neuromuscular disease in general, but will also provide unique insights into the pathophysiology of these disorders.

The study of muscle biopsies from FSHD patients means that the data acquired will reflect consequences of pathology in adult, differentiated human muscle cells. This prevents possible confounders that may arise with other frequently used methods in FSHD research such as myotubes, myoblasts or mouse models.

As in all studies that use biopsies, the sampled material represents only a small part of the muscle. This is a risk especially in FSHD, which is characterized by a marked difference in muscle pathology even within the same muscle. In this study, we will use MRI imaging of the area of muscle biopsy to provide information on atrophy, muscle/fat fraction and inflammation to correct for sampling error.

In conclusion, this study will provide new insights in sarcomeric function of patients suffering from FSHD, and correlate these to clinical measures in the same patients. This will be an important step in the understanding of the pathophysiology of muscle weakness in this disease, possibly identifying novel targets for therapeutic intervention.

## Competing interests

The authors declare that they have no competing interests.

## Authors’ contributions

BGME and CACO designed the study and obtained funding. HJW performed extensive preparative work prior to the start of this study and drafted the study protocol. SL is the primary investigator of this study and responsible for collection, analysis, and interpretation of the data, and wrote the manuscript. BHJ designed the MRI scanning protocol and supervises analysis and interpretation of MRI data. MTH supervises the collection and analysis of data for quantitative muscle studies. CACO and GJMS will supervise collection and analysis of data for contractile studies. CACO, NCV, BGME, GJMS, SMvdM and GWP will supervise the execution of the study and interpretation of the data. All authors read and approved the final manuscript.

## Pre-publication history

The pre-publication history for this paper can be accessed here:

http://www.biomedcentral.com/1471-2377/13/144/prepub

## References

[B1] Emery AE Population frequencies of inherited neuromuscular diseases–a world survey Neuromuscul Disord 1991 1 1 19 29 10.1016/0960-8966(91)90039-U 1822774

[B2] van der Maarel SM Frants RR Padberg GW Facioscapulohumeral muscular dystrophy Biochim Biophys Acta 2007 1772 2 186 194 10.1016/j.bbadis.2006.05.009 16837171

[B3] Padberg GW Facioscapulohumeral dystrophy 1982 Leiden, the, Netherlands: Leiden University

[B4] Pandya S King WM Tawil R Facioscapulohumeral dystrophy Phys Ther 2008 88 1 105 113 10.2522/ptj.20070104 17986494

[B5] Lemmers RJ van der Vliet PJ Klooster R Sacconi S Camano P Dauwerse JG Snider L Straasheijm KR van Ommen GJ Padberg GW A unifying genetic model for facioscapulohumeral muscular dystrophy Science 2010 329 5999 1650 1653 10.1126/science.1189044 20724583 PMC4677822

[B6] van der Maarel SM Tawil R Tapscott SJ Facioscapulohumeral muscular dystrophy and DUX4: breaking the silence Trends Mol Med 2011 17 5 252 258 10.1016/j.molmed.2011.01.001 21288772 PMC3092836

[B7] Lunt PW Jardine PE Koch MC Maynard J Osborn M Williams M Harper PS Upadhyaya M Correlation between fragment size at D4F104S1 and age at onset or at wheelchair use, with a possible generational effect, accounts for much phenotypic variation in 4q35-facioscapulohumeral muscular dystrophy (FSHD) Hum Mol Genet 1995 4 5 951 958 10.1093/hmg/4.5.951 7633457

[B8] de Greef JC Lemmers RJ Camano P Day JW Sacconi S Dunand M van Engelen BG Kiuru-Enari S Padberg GW Rosa AL Clinical features of facioscapulohumeral muscular dystrophy 2 Neurology 2010 75 17 1548 1554 10.1212/WNL.0b013e3181f96175 20975055 PMC2974464

[B9] Lemmers RJ Tawil R Petek LM Balog J Block GJ Santen GW Amell AM van der Vliet PJ Almomani R Straasheijm KR Digenic inheritance of an SMCHD1 mutation and an FSHD-permissive D4Z4 allele causes facioscapulohumeral muscular dystrophy type 2 Nature genetics 2012 44 12 1370 1374 10.1038/ng.2454 23143600 PMC3671095

[B10] Du J Wang X Miereles C Bailey JL Debigare R Zheng B Price SR Mitch WE Activation of caspase-3 is an initial step triggering accelerated muscle proteolysis in catabolic conditions J Clin Invest 2004 113 1 115 123 14702115 10.1172/JCI200418330 PMC300763

[B11] Wallace LM Garwick SE Mei W Belayew A Coppee F Ladner KJ Guttridge D Yang J Harper SQ DUX4, a candidate gene for facioscapulohumeral muscular dystrophy, causes p53-dependent myopathy in vivo Annals Neurology 2011 69 3 540 552 10.1002/ana.22275 PMC4098764 21446026

[B12] Geng LN Yao Z Snider L Fong AP Cech JN Young JM van der Maarel SM Ruzzo WL Gentleman RC Tawil R DUX4 activates germline genes, retroelements, and immune mediators: implications for facioscapulohumeral dystrophy Dev Cell 2012 22 1 38 51 10.1016/j.devcel.2011.11.013 22209328 PMC3264808

[B13] Vanderplanck C Ansseau E Charron S Stricwant N Tassin A Laoudj-Chenivesse D Wilton SD Coppee F Belayew A The FSHD atrophic myotube phenotype is caused by DUX4 expression PLoS One 2011 6 10 e26820 10.1371/journal.pone.0026820 22053214 PMC3203905

[B14] Lokireddy S McFarlane C Ge X Zhang H Sze SK Sharma M Kambadur R Myostatin induces degradation of sarcomeric proteins through a Smad3 signaling mechanism during skeletal muscle wasting Mol Endocrinol 2011 25 11 1936 1949 10.1210/me.2011-1124 21964591 PMC5417176

[B15] Liu Q Jones TI Tang VW Brieher WM Jones PL Facioscapulohumeral muscular dystrophy region gene-1 (FRG-1) is an actin-bundling protein associated with muscle-attachment sites J Cell Sci 2010 123 Pt 7 1116 1123 20215405 10.1242/jcs.058958 PMC2844320

[B16] Hanel ML Sun CY Jones TI Long SW Zanotti S Milner D Jones PL Facioscapulohumeral muscular dystrophy (FSHD) region gene 1 (FRG1) is a dynamic nuclear and sarcomeric protein Differ res Biol Diversity 2011 81 2 107 118 10.1016/j.diff.2010.09.185 PMC3030934 20970242

[B17] Gabellini D D'Antona G Moggio M Prelle A Zecca C Adami R Angeletti B Ciscato P Pellegrino MA Bottinelli R Facioscapulohumeral muscular dystrophy in mice overexpressing FRG1 Nature 2006 439 7079 973 977 16341202 10.1038/nature04422

[B18] Wuebbles RD Hanel ML Jones PL FSHD region gene 1 (FRG1) is crucial for angiogenesis linking FRG1 to facioscapulohumeral muscular dystrophy-associated vasculopathy Disease Models Mechanisms 2009 2 5–6 267 274 19383939 10.1242/dmm.002261 PMC2675802

[B19] Lassche S Stienen GJ Irving TC van der Maarel SM Voermans NC Padberg GW Granzier H van Engelen BG Ottenheijm CA Sarcomeric dysfunction contributes to muscle weakness in facioscapulohumeral muscular dystrophy Neurology 2013 80 8 733 737 10.1212/WNL.0b013e318282513b 23365058 PMC3589299

[B20] Hilton-Jones D Miller A Parton M Holton J Sewry C Hanna MG Inclusion body myositis: MRC Centre for Neuromuscular Diseases, IBM workshop, London, 13 June 2008 Neuromuscular Disord: NMD 2010 20 2 142 147 10.1016/j.nmd.2009.11.003 20074951

[B21] Mathieu J Boivin H Meunier D Gaudreault M Begin P Assessment of a disease-specific muscular impairment rating scale in myotonic dystrophy Neurology 2001 56 3 336 340 10.1212/WNL.56.3.336 11171898

[B22] Ricci E Galluzzi G Deidda G Cacurri S Colantoni L Merico B Piazzo N Servidei S Vigneti E Pasceri V Progress in the molecular diagnosis of facioscapulohumeral muscular dystrophy and correlation between the number of KpnI repeats at the 4q35 locus and clinical phenotype Annals Neurology 1999 45 6 751 757 10.1002/1531-8249(199906)45:6<751::AID-ANA9>3.0.CO;2-M 10360767

[B23] van Overveld PG Enthoven L Ricci E Rossi M Felicetti L Jeanpierre M Winokur ST Frants RR Padberg GW van der Maarel SM Variable hypomethylation of D4Z4 in facioscapulohumeral muscular dystrophy Annals Neurology 2005 58 4 569 576 10.1002/ana.20625 16178028

[B24] Kalkman JS Schillings ML Zwarts MJ van Engelen BG Bleijenberg G The development of a model of fatigue in neuromuscular disorders: a longitudinal study J Psychosom Res 2007 62 5 571 579 10.1016/j.jpsychores.2006.11.014 17467412

[B25] van der Werf SP Prins JB Vercoulen JH van der Meer JW Bleijenberg G Identifying physical activity patterns in chronic fatigue syndrome using actigraphic assessment J Psychosom Res 2000 49 5 373 379 10.1016/S0022-3999(00)00197-5 11164063

[B26] Fries JF Spitz P Kraines RG Holman HR Measurement of patient outcome in arthritis Arthritis and rheumatism 1980 23 2 137 145 10.1002/art.1780230202 7362664

[B27] Craig CL Marshall AL Sjostrom M Bauman AE Booth ML Ainsworth BE Pratt M Ekelund U Yngve A Sallis JF International physical activity questionnaire: 12-country reliability and validity Med Sci Sports Exerc 2003 35 8 1381 1395 10.1249/01.MSS.0000078924.61453.FB 12900694

[B28] Jacobs HM Luttik A Touw Otten FW De Melker RA The sickness impact profile; results of an evaluation study of the Dutch version] Nederlands tijdschrift voor geneeskunde 1990 134 40 1950 1954 2234151

[B29] Vanderiet K Adriaensen H Carton H Vertommen H The McGill Pain Questionnaire constructed for the Dutch language (MPQ-DV). Preliminary data concerning reliability and validity Pain 1987 30 3 395 408 10.1016/0304-3959(87)90027-3 3670884

[B30] Council MR Aids to the examination of the peripheral nervous system, Memorandum no 45. edn 1981 London, UK: Her Majesty's Stationery Office

[B31] Berard C Payan C Hodgkinson I Fermanian J A motor function measure for neuromuscular diseases, construction and validation study Neuromuscul Disord 2005 15 7 463 470 10.1016/j.nmd.2005.03.004 16106528

[B32] Enright PL The six-minute walk test Respir Care 2003 48 8 783 785 12890299

[B33] Podsiadlo D Richardson S The timed "Up & Go": a test of basic functional mobility for frail elderly persons J Am Geriatr Soc 1991 39 2 142 148 1991946 10.1111/j.1532-5415.1991.tb01616.x

[B34] Degens H Sanchez Horneros JM Heijdra YF Dekhuijzen PN Hopman MT Skeletal muscle contractility is preserved in COPD patients with normal fat-free mass Acta physiologica Scandinavica 2005 184 3 235 242 10.1111/j.1365-201X.2005.01447.x 15954991

[B35] Wattjes MP Kley RA Fischer D Neuromuscular imaging in inherited muscle diseases Eur Radiol 2010 20 10 2447 2460 10.1007/s00330-010-1799-2 20422195 PMC2940021

[B36] Kan HE Scheenen TW Wohlgemuth M Klomp DW van Loosbroek-Wagenmans I Padberg GW Heerschap A Quantitative MR imaging of individual muscle involvement in facioscapulohumeral muscular dystrophy Neuromuscul Disord 2009 19 5 357 362 10.1016/j.nmd.2009.02.009 19329315

[B37] The FSH-DY Group A prospective, quantitative study of the natural history of facioscapulohumeral muscular dystrophy (FSHD): implications for therapeutic trials Neurology 1997 48 1 38 46 9008491 10.1212/wnl.48.1.38

[B38] Ottenheijm CA Hooijman P DeChene ET Stienen GJ Beggs AH Granzier H Altered myofilament function depresses force generation in patients with nebulin-based nemaline myopathy (NEM2) J Struct Biol 2010 170 2 334 343 10.1016/j.jsb.2009.11.013 19944167 PMC2856782

[B39] Stienen GJ Kiers JL Bottinelli R Reggiani C Myofibrillar ATPase activity in skinned human skeletal muscle fibres: fibre type and temperature dependence J Physiol 1996 493 Pt 2 299 307 8782097 10.1113/jphysiol.1996.sp021384 PMC1158918

[B40] Ottenheijm CA Lawlor MW Stienen GJ Granzier H Beggs AH Changes in cross-bridge cycling underlie muscle weakness in patients with tropomyosin 3-based myopathy Hum Mol Genet 2011 20 10 2015 2025 10.1093/hmg/ddr084 21357678 PMC3080611

[B41] Chandra M Mamidi R Ford S Hidalgo C Witt C Ottenheijm C Labeit S Granzier H Nebulin alters cross-bridge cycling kinetics and increases thin filament activation: a novel mechanism for increasing tension and reducing tension cost J Biol Chem 2009 284 45 30889 30896 10.1074/jbc.M109.049718 19736309 PMC2781488

[B42] Ottenheijm CA Witt CC Stienen GJ Labeit S Beggs AH Granzier H Thin filament length dysregulation contributes to muscle weakness in nemaline myopathy patients with nebulin deficiency Hum Mol Genet 2009 18 13 2359 2369 10.1093/hmg/ddp168 19346529 PMC2694687

[B43] Ottenheijm CA Knottnerus AM Buck D Luo X Greer K Hoying A Labeit S Granzier H Tuning passive mechanics through differential splicing of titin during skeletal muscle development Biophys J 2009 97 8 2277 2286 10.1016/j.bpj.2009.07.041 19843460 PMC2764098

[B44] Warren CM Krzesinski PR Greaser ML Vertical agarose gel electrophoresis and electroblotting of high-molecular-weight proteins Electrophoresis 2003 24 11 1695 1702 10.1002/elps.200305392 12783444

[B45] Ottenheijm CA Heunks LM Geraedts MC Dekhuijzen PN Hypoxia-induced skeletal muscle fiber dysfunction: role for reactive nitrogen species Am J Physiol Lung Cell Mol Physiol 2006 290 1 L127 L135 16113049 10.1152/ajplung.00073.2005

[B46] Ottenheijm CA Heunks LM Sieck GC Zhan WZ Jansen SM Degens H de Boo T Dekhuijzen PN Diaphragm dysfunction in chronic obstructive pulmonary disease Am J Respir Crit Care Med 2005 172 2 200 205 10.1164/rccm.200502-262OC 15849324 PMC2718467

[B47] Lahmers S Wu Y Call DR Labeit S Granzier H Developmental control of titin isoform expression and passive stiffness in fetal and neonatal myocardium Circ Res 2004 94 4 505 513 10.1161/01.RES.0000115522.52554.86 14707027

